# PHF6 promotes the progression of endometrial carcinoma by increasing cancer cells growth and decreasing T‐cell infiltration

**DOI:** 10.1111/jcmm.17638

**Published:** 2023-02-08

**Authors:** Xiaomin Wang, Aizhong Fang, Yichen Peng, Jianyu Yu, Chunna Yu, Jinxuan Xie, Yi Zheng, Lairong Song, Parker Li, Jia Li, Xun Kang, Yi Lin, Wenbin Li

**Affiliations:** ^1^ Cancer Center, Beijing Tiantan Hospital Capital Medical University Beijing China; ^2^ Department of Epidemiology and Health Statistics, School of Public Health Capital Medical University Beijing China; ^3^ Clinical Medicine Shanghai Jiao Tong University School of Medicine Shanghai China; ^4^ Department of Pathology, Beijing Tiantan Hospital Capital Medical University Beijing China

**Keywords:** cell cycle, clinical outcome, epigenetic regulated gene, gene regulation, immune infiltration, PHF6, uterine corpus endometrial carcinoma

## Abstract

Uterine corpus endometrial carcinoma (UCEC) is the most common cancer of the female reproductive tract. The overall survival of advanced and recurrent UCEC patients is still unfavourable nowadays. It is urgent to find a predictive biomarker and block tumorgenesis at an early stage. Plant homeodomain finger protein 6 (PHF6) is a key player in epigenetic regulation, and its alterations lead to various diseases, including tumours. Here, we found that PHF6 expression was upregulated in UCEC tissues compared with normal tissues. The UCEC patients with high PHF6 expression had poor survival than UCEC patients with low PHF6 expression. *PHF6* mutation occurred in 12% of UCEC patients, and *PHF6* mutation predicted favourable clinical outcome in UCEC patients. Depletion of PHF6 effectively inhibited HEC‐1‐A and KLE cell proliferation in vitro and decreased HEC‐1‐A cell growth in vivo. Furthermore, high PHF6 level indicated a subtype of UCECs characterized by low immune infiltration, such as CD3^+^ T‐cell infiltration. While knockdown of PHF6 in endometrial carcinoma cells increased T‐cell migration by promoting IL32 production and secretion. Taken together, our findings suggested that PHF6 might play an oncogenic role in UCEC patients. Thus, PHF6 could be a potential biomarker in predicting the prognosis of UCEC patients. Depletion of PHF6 may be a novel therapeutic strategy for UCEC patients.

## 1 INTRODUCTION

Solid tumours are prevalent in which it is estimated of about 80% share of all tumour types just for a subset of solid organs, and the share of solid tumour in cancer‐related death is more than 85%.^1^ Cancer cell transformation occurs through the accumulation of different genomic and epigenomic gene alterations.^2^, ^3^, ^4^ The complexity and multifactorial nature of cancer are part of the most significant challenges in the diagnosis and treatment of cancer patients. Within the past decade, accumulating knowledge about the epigenetic basis of tumorgenesis revolutionized the field of cancer genetics in the postgenomic era, providing novel biomarkers and new targets for cancer therapy.^3^, ^4^ Plant homeodomain finger protein 6 (PHF6) is a significant epigenetic regulator located on the X chromosome. Its mutations are often implicated in haematological malignancies, especially in T‐lymphoblastic leukaemia and acute myeloid leukaemia.^5^ Interestingly, our recent studies showed that PHF6 mutations frequently occurred in uterine corpus endometrial carcinoma (UCEC) patients, and PHF6 mutation predicted a favourable prognosis for UCEC patients. However, the functional role(s) of PHF6 in UCEC initiation, maintenance and progression are currently unknown.

Uterine corpus endometrial carcinoma is the most common cancer of the female reproductive tract.^6^ It often affects postmenopausal women and has an incidence peak at the median diagnosis age of 63.^7^ Even though 75% of UCEC patients are diagnosed at an early stage owing to the obvious clinical presentation of abnormal vaginal bleeding, the overall survival (OS) of patients with advanced and recurrent UCEC remains unfavourable.^8^ Many efforts have been dedicated to deciphering the molecular events underlying endometrial carcinogenesis, with the goals of identifying specific therapeutic targets and developing new and more effective drugs. In this study, we focused on the biological role of PHF6 in carcinogenesis in UCEC by using data from multiple databases. We found high level of PHF6 predicted the poor survival of UCEC patients. PHF6 might promote UCEC progression by increasing proliferation of cancer cells and reducing migration of T cells. PHF6 might play an oncogenic role in UCEC and thus potentially be a therapeutic target for treating UCEC patients.

## 2 MATERIALS AND METHODS

### 2.1 Data acquisition

**FIGURE 1:**
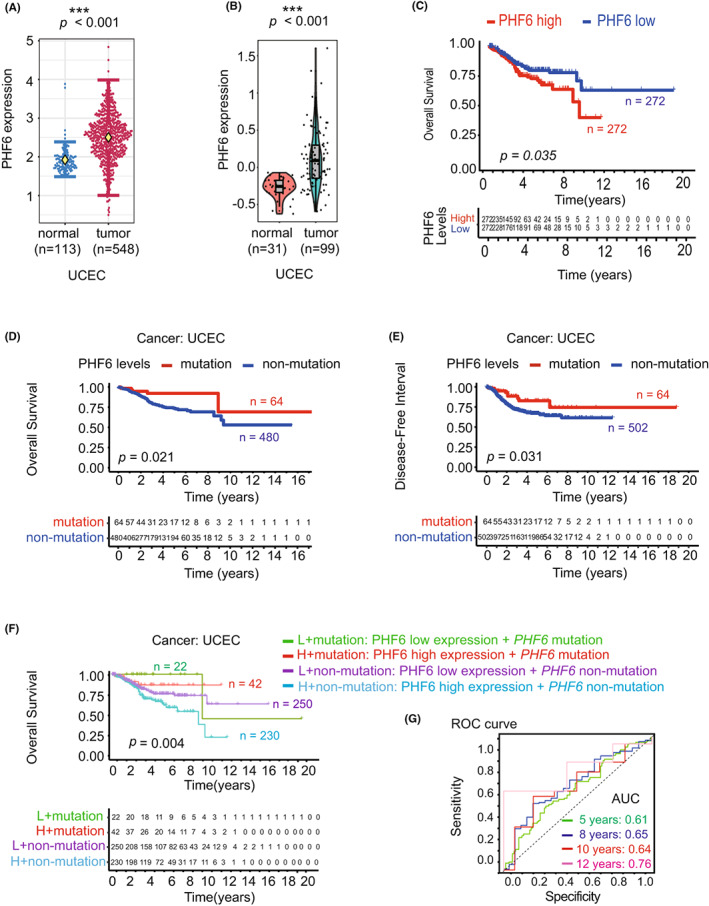
High expression of PHF6 predicted the unfavourable survival of UCEC patients. (A) The mRNA expression of PHF6 in UCEC tissues and normal tissues from the TCGA and the GTEx database. (B) The protein expression of PHF6 in tumour tissues and normal tissues in UCEC patients from the TCGA database. (C) Kaplan–Meier survival curves showing the overall survival (OS) of UCEC patients with high PHF6 expression or UCEC patients with low PHF6 expression. (D‐E) The correlation between *PHF6* mutation status and OS or progression‐free interval (PFI) of UCEC patients from TCGA dataset. (F) The overall survival curve of UCEC patients with low‐PHF6 + *PHF6* mutation, high‐PHF6 + *PHF6* mutation, low‐PHF6 + *PHF6* non‐mutation, or high‐PHF6 + *PHF6* non‐mutation. (G) The ROC curve for predicting the efficacy of PHF6 for UCEC patients. AUC at 5 years of 0.61, 8 years of 0.65, 10 years of 0.64, and 12 years of 0.76. (* represents a significant difference, * *p* < 0.05)

We firstly collected the mRNA expression profiles of UCECs and survival information from the The Cancer Genome Atlas (TCGA)‐UCECs datasets in Figure 1A,C. The mRNA expression profiles of normal control were from TCGA‐UCECs and Genotype‐Tissue Expression (GTEx)‐uterus dataset in Figure 1A. We collected the protein expression profiles of UCECs and normal control from CPTAC‐UCECs database in Figure 1B. We collected the PHF6 mutation and survival information in UCEC patients from TCGA‐UCECs dataset in Figure 1D,E. All the patients' IDs are listed in Appendix S4.

### 2.2 Survival analysis

In order to investigate the relationship between PHF6 expression level and the prognosis of patients, we use the Kaplan–Meier (KM) method by the survival package in R Version 4.1.1. For further verification, we performed OS and Relapse free survival (RFS) analyses based on PHF6 expression levels by Kaplan–Meier Plotter (http://kmplot.com/analysis/).

### 2.3 GO enrichment and netplot

First, we analysed the differentially expressed genes (DEGs) between PHF6‐high and PHF6‐low groups. Gene ontology (GO) was used to explore potential functions of PHF6 in UCEC. The package in R to complete the above research is cluster Profiler. Finally, with the DEGs above, we utilized Cytoscape to capture some immune‐related GO terms in the form of a Netplot.

### 2.4 Immune infiltration

For the purpose of exploring the correlation between PHF6 expression and immune cells, we analysed the immune score and stromal score of each tumour sample in UCEC by the package ESTIMATE and CIBERSORT. Moreover, we divided the samples into PHF6‐high and PHF6‐low groups according to the expression level to observe the differences in the abundance of 22 immune fractions with the R package CIBERSORT. The Wilcoxon rank‐sum test was used to capture the differences.

### 2.5 Immunohistochemical analysis

The tissue microarray (TMA) was purchased from Shanghai Outdo Biotech Co., Ltd. The tissues for immunohistochemical analysis were fixed with formalin, followed by embedment in paraffin. After being dewaxed in xylene and ethyl alcohol, antigen retrieval and being rinsed with PBS, we incubated the slides with anti‐CD3 antibody (85061T, 1:300, Abcam), anti‐CD4 antibody (ab288724, 1:1000, Abcam), anti‐CD8 antibody (ab245118, 1:600, Abcam), anti‐CD19 antibody (90176, 1:300, Abcam), anti‐CD33 antibody (ab269456, 1:200, Abcam), anti‐CDK4 antibody (108357, 1:300, Abcam) and anti‐PHF6 antibody (ab264208, 1:500, Abcam) overnight at four degrees Celsius and then at indoor temperature for half an hour, and then incubated with an anti‐rabbit/mouse IgG H&L antibody (GK500710, Genetech, China) at indoor temperature for 1 h and then incubated with a DAB Substrate Kit (1:20) for 10 min, counterstaining with haematoxylin for 15 min, differentiation fluid for 15 s and return blue liquid for 1 min. The slide was rehydrated with graded concentrations of ethanol, cleared with xylene, dried and sealed.

The staining intensity of whole tumour tissue was estimated for the abundance of PHF6 and CDK4. We divided the staining intensity of whole tumour tissue into three levels, represented by 0–3, respectively. 0–1 symbolized the most weakly stained samples, while 3 represented the strongest intensity. For CD3/CD4/CD8/CD19/CD33 staining, the percentage of cells with strong intensity (brown staining) of membrane staining was only evaluated. Likewise, the degree of positive expression was classified into two groups with the division of samples with 40% positive expression, symbolized by 1 and 2, respectively. PHF6, CD3 and CDK4 staining was independently evaluated for immunoreactivity by two pathologists (Tables S2 and S3).

### 2.6 Cell lines and culture

HEC‐1‐A and KLE cells were obtained from the American Type Culture Collection (ATCC). The cells were identified by STR profiling. HEC‐1‐A and KLE cells were cultured in high glucose DMEM (Corning) supplemented with 10% FBS, 1% penicillin/streptomycin and 1% l‐Glutamine. Trypsin/EDTA (0.25%) was used for dissociation. The two different miRNA oligos corresponding to PHF6 and the scrambled negative‐control miRNA were purchased from Invitrogen. The siRNA was transfected into HEC‐1‐A and KLE cells by Lipofectamine™ 2000 (Invitrogen, 11668‐027). The PHF6 mRNA was detected by real‐time PCR 24 h after transfection with siRNA. The PHF6 protein was detected by Western blot 48 hours after transfection with siRNA. The siRNA oligos for PHF6 KD‐1: GGTCAGTTTCTCATGAAGATA. The siRNA oligos for PHF6 KD‐2: GACAAAATGGAGACATTGATA.

### 2.7 Plasmid construction

PCR primers were designed to amplify the full length of CDK4 with the addition of a 5′ BamHI and a 3′ KpnI site. Primers were as follows: Forward, 5′‐CACACTGGACTAGTGGATCCCGCCACCATGGCTACCTCTCGATATGAG‐3′; Reverse, 5′‐AGTCACTTAAGCTTGGTACCTCCGGATTACCTTCATCCTTATG‐3′. Using standard molecular biology techniques, the PCR product was generated and ligated into GV208 vector (Genechem).

### 2.8 Mouse model

To investigate the role of PHF6 in the proliferation of HEC‐1‐A cells, PHF6 KD HEC‐1‐A cells (5 × 10^5^) were mixed with 100 μl matrigel and implanted subcutaneously into 8‐week‐old female NOD/SCID/IL‐2Rγnull (NSG) mice. Tumour volume was measured every 3 days. To investigate the role of PHF6 KD HEC‐1‐A cells in the infiltration of T cells, the T cells (1 × 10^6^) were injected into NSG mice 1 week after implantation with PHF6 KD HEC‐1‐A cells (5 × 10^5^). The mice were sacrificed 3 weeks after implantation with HEC‐1‐A cells. The animal experiments were approved by the Institutional Animal Care and Use Committee of Capital Medical University (Permit Number: CMU07802161).

### 2.9 Cell proliferation assay

We harvested the cells 48 h after transfection with siRNA. Then, we evaluated the growth ability of PHF6 KD cells and control cells. 3 × 10^3^ PHF6 KD and control cancer cells were seeded per well in 96‐well plates. Cells were plated in five wells per time point. The wells were incubated with 10 μl CCK‐8 (Sigma‐Aldrich) for 2 h in 37°C cell culture incubator. Then, the absorbance was measured at 450 nm by Paradigm Detection Platform (BECKMAN). The growth curve of cancer cells was plotted in Graphpad Prism 5.0.

### 2.10 Cell cycle analysis

Cells were fixed using the Cytofix Fixation/Permeabilization Kit (BD Biosciences) according to the manufacturer's instructions. Cells were stained with Ki67 antibody at room temperature for 30 min. Prior to analysis, cells were incubated with 1.62 μM Hochest 33342 (Invitrogen) at room temperature. Cells were analysed using a FACS Canto flow cytometer (BD Biosciences). Data were analysed with FlowJo software (Tree Star).

### 2.11 Apoptosis assay

PHF6 KD and control cancer cells were used for analysis. The apoptosis of cells was examined using the Annexin‐V/PI Apoptosis Detection Kit (BD Biosciences) according to manufacturer's instructions.

### 2.12 T cells migration assay

T‐cell migration assay was performed using 8‐μm pore size cell culture insert in a 24‐well plate (BD Falcon). T cells were sorted from healthy donors. 1 × 10^5^ T cells were seeded into the top of the transwell chambers, and PHF6 KD or control cancer cells were seeded in the lower chambers. After 6 h of incubation, cells in the upper membrane surface were removed with cotton swab. Cells on the lower membrane surface were stained with 0.1% Crystal Violet for 30 min at room temperature. The cells in the lower chambers were counted by Countess II (Invitrogen).

### 2.13 IL32 neutralizing experiment

For the IL32 neutralization experiment, IL32 antibody (R&D, AF3040) was added to the culture system at 10 ng/ml. 1 × 10^5^ T cells were seeded into the top of the transwell chambers, and PHF6 KD or control cancer cells were seeded in the lower chambers. After 6 h of incubation, cells in the upper membrane surface were removed with cotton swab. Cells on the lower membrane surface were stained with 0.1% Crystal Violet for 30 min at room temperature. The cells in the lower chambers were counted by Countess II (Invitrogen).

### 2.14 Quantitative real‐time PCR

Total RNA was extracted from cells using TRIzol Reagent (Life Technologies, USA) according to manufacturer's instructions. cDNA was synthesized according to manufacturer's instructions of Revert Aid First SYBR Green PCR Kit (Thermo Fisher Scientific) with one μg RNA. PCR reactions were run with the CFX96 real‐time PCR detection system (Bio‐Rad, USA) according to the protocol of 2× SYBR Green qPCR Master Mix (Bimake). Primers used for PCR were as follows: human PHF6 F‐TGCTTTGGTGTCCTCACATTCT, R‐GCGAAGGTTTCTCTCGGATCT; human GAPDH F‐TTAAAAGCAGCCCTGG, R‐GACAGTCAGCCGCATCTTCT. GAPDH was used as a control. human CDK4 F‐CCATAGGCACCGACACCAAT, R‐GCGTGAGGGTCTCCCTTG; human CDK7 F‐TAACGCTTTGCCCGAGACTT; R‐ATTCGTGTTGTCCTGGGA.

### 2.15 Western blot

The cells were harvested 48 h after transfection with siRNA. Total protein was extracted using RIPA with 1% PMSF (Solarbio). 30–90 μg protein was separated using 10% SDS–PAGE and then transferred to PVDF membranes (Millipore, MA). PVDF membranes were blocked with milk for 1 h at indoor temperature and incubated with primary antibody overnight at four degrees Celsius. Then, PVDF membranes were incubated in secondary antibody (1:2000) (Zsbio) at indoor temperature for 1 h. Finally, ECL detection method (Bio‐Rad) was used for imaging analysis. Antibody list: PHF6‐antibody (Abcam, ab173304), CDK4‐antibody (Abcam, ab108357) and Actin‐antibody (Abcam, ab8226).

### 2.16 ELISA

The ELISA was performed using the human IL32 and the human IL12 ELISA Kit according to the manufacturer's protocols (Anoric, TAE‐327 h and TAE‐466 h, China). 5 × 10^5^ cells in 200 μl of water were frozen and thawed three times and centrifuged at 3000*g* (5,915 rpm) for 5 min, and the liquid supernatant was collected for IL32 and IL12 test. In addition, the culture medium was collected for IL32 and IL12 tests. The ELISA was read on a SynergyH 4 Hybrid Reader at 450 nm.

### 2.17 Statistical analysis

Statistical analysis and graphical visualization were completed by R software (Version 4.1.1). We explored the expression of PHF6 between tumours and normal tissues through Wilcoxon rank‐sum test, and *p* < 0.05 was regarded as statistically significant By log‐rank tests, we compared survival curves between PHF6‐high and PHF6‐low groups. Pearson analysis was applied to estimate the connection between the *PHF6* expression levels and immune cells. Student's two‐tailed *t*‐test and one‐way anova are used in Figures 3 and 4. We regarded the *p*‐value <0.05 as statistically significant (*p < 0.05; ** p < 0.01; *** p < 0.001).

## 3 RESULTS

### 3.1 High expression of PHF6 is associated with unfavourable prognosis of UCEC patients

To investigate the role of the epigenetic gene PHF6 in tumours, we systematically analysed the mRNA expression of PHF6 in 33 different tumour types from TCGA and GTEx dataset through Xena (https://xenabrowser.net/) analysis system, which included 10,237 patients (Figure S1A). We found that the mRNA level of PHF6 was significantly upregulated in UCEC tissues compared with their corresponding normal controls (Figure 1A and Figure S1A). In addition, the protein expression of PHF6 was also much higher in UCEC tissues than in normal controls from CPTAC database (Figure 1B). To understand the relationship between the expression level of PHF6 and the clinical outcome, UCEC patients were grouped into low‐PHF6‐group and high‐PHF6‐group according to the median mRNA expression of PHF6 for further analysis by R 4.1.1. for overall survival (OS). We found that the high expression of PHF6 was significantly associated with poor survival of UCEC patients (*p* = 0.035), indicating that PHF6 might involve in the carcinogenesis of UCECs (Figure 1C). Furthermore, we explored the potential relationship between genetic alterations of *PHF6* and the survival of UCEC patients from TCGA dataset. As exhibited in Figure S1B, *PHF6* mutation occurred in 12% of UCEC patients (Figure S1B). Uterine corpus endometrial carcinoma patients with *PHF6* mutations had a favourable prognosis in terms of OS (*p* = 0.021) and DFI (*p* = 0.031) compared with patients without *PHF6* mutations (Figure 1D,E). Uterine corpus endometrial carcinoma patients were grouped into low‐PHF6 + *PHF6* mutation (L + mutation), high‐PHF6 + *PHF6* mutation (H + mutation), low‐PHF6 + *PHF6* non‐mutation (L + non‐mutation) and high‐PHF6 + *PHF6* non‐mutation (H + non‐mutation) for further analysis of overall survival (OS). Interestingly, we found that the UCEC patients with the low‐PHF6 + *PHF6* mutation had the best survival, while the high‐PHF6 + *PHF6* wild‐type group had the worst survival in four groups (*p* = 0.004). The survival status of the high‐PHF6 + *PHF6* mutation group was much better than low‐PHF6 + *PHF6* wild‐type group (Figure 1F). Then, we plotted the ROC curve to predict efficacy of PHF6 for UCEC patients. The ROC curve showed a modest diagnostic value with AUC at 5 years of 0.61, 8 years of 0.65, 10 years of 0.64, and at 12 years of 0.76 (Figure 1G). These results suggested that *PHF6* might be a potential biomarker in predicting the clinical outcomes of UCEC patients.

Additionally, we analysed the correlations of PHF6 mRNA expression with clinical characteristics in UCECs from TCGA database, including age (*n* = 541), clinical stage (*n* = 544), histological grade (*n* = 544) and tumour status (*n* = 523). We found that the mRNA expression of PHF6 was related to the histological grade (*p* = 0.0004) (Table S1).

### 3.2 Depletion of PHF6 inhibits the growth of endometrial carcinoma cells by blocking cell cycle in vivo and in vitro

**FIGURE 2:**
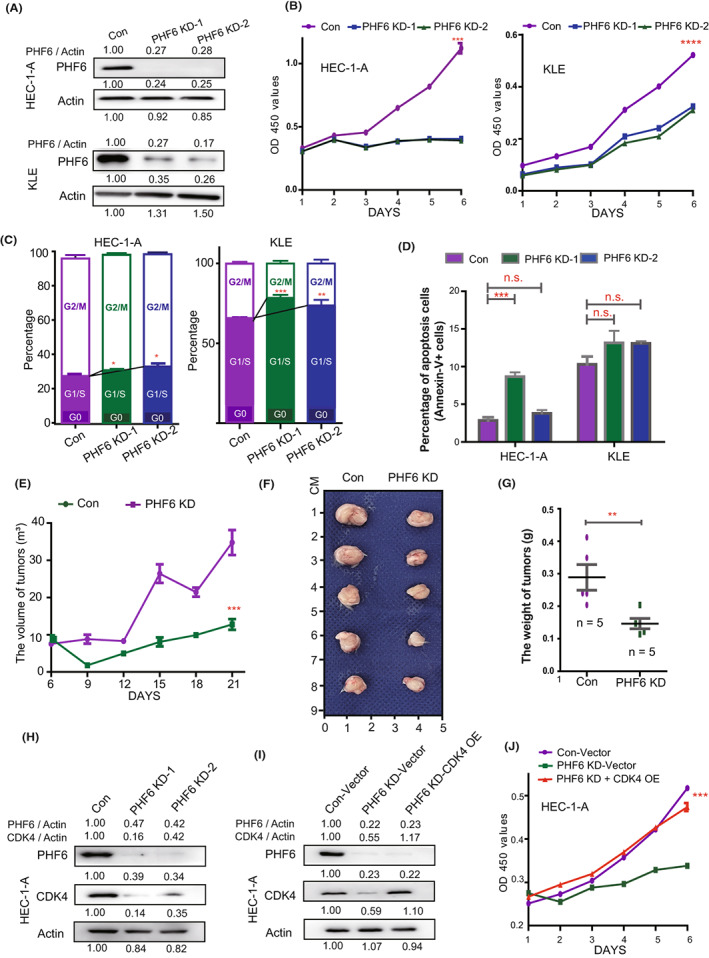
Knockdown of PHF6 inhibited the growth of endometrial carcinoma cells by blocking CDK4 expression in vitro and in vivo. (A) Up panel, the expression of PHF6 in PHF6 KD HEC‐1‐A and control cells. Down panel, the expression of PHF6 in PHF6 KD KLE and control cells. (B) The growth curve of PHF6 KD endometrial carcinoma cells and control cells. (C) The cell cycle stage of PHF6 KD endometrial carcinoma cells and control cells. (D) The apoptosis level of PHF6 KD endometrial carcinoma cells and control cells. (E) The growth of PHF6 KD HEC‐1‐A or control cells subcutaneously xenografted tumours in female NSG mice (*n* = 5). Tumour volume was measured every 3 days. (F) The tumour mass of PHF6 KD HEC‐1‐A or control cells subcutaneously xenografts after harvest. (G) The weight of tumours harvested from NSG mice. (H) The protein expression of CDK4 and PHF6 in PHF6 KD HEC‐1‐A or control cells. (I) The protein expression of PHF6 and CDK4 in Con‐Vector, PHF6 KD‐Vector and PHF6 KD‐CDK4 OE HEC‐1‐A cells. (J) The growth curve of Con‐Vector, PHF6 KD‐Vector and PHF6 KD‐CDK4 OE HEC‐1‐A cells.

To further determine the role of PHF6 in carcinogenesis of UCECs, we knocked down PHF6 expression by transfecting with siRNA targeting PHF6. Knockdown of PHF6 (PHF6 KD) was validated by Western blot and real‐time PCR (Figure 2A and Figure S2A). In growth curve assay, knockdown of PHF6 led to a decrease in cell proliferation in HEC‐1‐A and KLE endometrial carcinoma cells (Figure 2B). We next performed DNA quantification with Hoechest 33342 or propidium iodide (PI) to evaluate the effect of PHF6 KD on cell cycle in HEC‐1‐A or KLE cells, respectively. PHF6 KD‐1 and PHF6 KD‐2 increased the G1/S ratio and decreased the G2/M ratio in HEC‐1‐A and KLE cells, suggesting that the change in proliferation rate might be caused by G1/S cell cycle arrest (Figure 2C and Figure S2B–D). In HEC‐1‐A, PHF6 KD‐1 promoted the apoptosis of cells (*p* < 0.001), while PHF6 KD‐2 did not change the apoptosis of cells (*p* > 0.05; n.s.). In KLE, PHF6 KD‐1 and PHF6 KD‐2 did not change the apoptosis of cells (*p* > 0.05; n.s.) (Figure 2D and Figure S2E). It suggested that PHF6 KD‐1‐induced apoptosis in HEC‐1‐A was not the key cause of the lower growth of endometrial carcinoma cells.

To determine the effect of PHF6 KD in endometrial carcinoma cells in vivo, we implanted PHF6 KD HEC‐1‐A cells subcutaneously into the immunodeficient NOD SCID gamma mice (NSG). Knockdown of PHF6 significantly decreased tumour growth rate in vivo (Figure 2E), and the weight of PHF6 KD tumours was much lower than that of the controls (Figure 2F,G). To further investigate the underlying mechanism of PHF6 in tumour cell cycle, we analysed the mRNA expression of CDK1‐7 (cell cyclin‐dependent kinases) in PHF6 KD HEC‐1‐A cells. We found that the mRNA expression of CDK4 and CDK7 was much higher in HEC‐1‐A cells than that of the other CDKs (not shown). Notably, PHF6 KD significantly decreased the protein and mRNA expression of CDK4 (Figure 2H and Figure S2F), while PHF6 KD did not change the expression of CDK7 in HEC‐1‐A cells. Then, we detected the protein expression of PHF6 and CDK4 in tumours from mice implanted with PHF6 KD or control HEC‐1‐A cells. We found that CDK4 and PHF6 were decreased in tumours from mice implanted with PHF6 KD cells compared with controls (Figure S2G).

Furthermore, we over‐expressed CDK4 (CDK4 OE) in PHF6 KD HEC‐1‐A cells and detected cell proliferation (Figure 2I,J). We found that CDK4 OE could rescue the growth of PHF6 KD HEC‐1‐A cells compared with the control group (Figure 2J). These data suggested that PHF6 might regulate the proliferation of endometrial carcinoma cells through CDK4 signalling pathway. Depletion of PHF6 might delay the growth of endometrial carcinoma cells through decreasing CDK4 expression.

### 3.3 Depletion of PHF6 in endometrial carcinoma cells promotes infiltration of T cells

**FIGURE 3:**
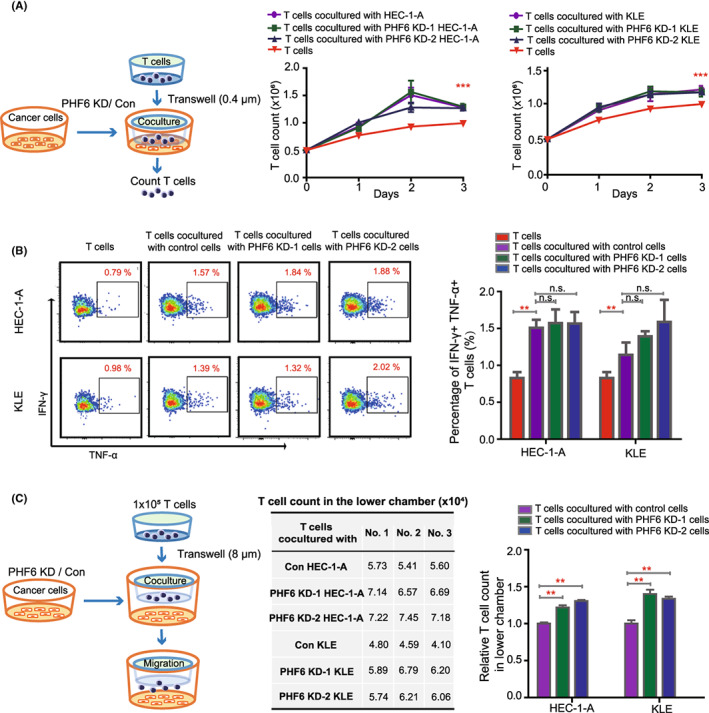
Knockdown of PHF6 in endometrial carcinoma cells promoted T‐cell migration. (A) Left panel, schematic representation of different endometrial carcinoma cells cocultured with T cells. Right panel, the growth curve of T cells cocultured with PHF6 KD endometrial carcinoma cells or control cells. (B) The percentage of IFN‐γ^+^ and TNF‐α^+^ cells in T cells when cocultured with PHF6 KD endometrial carcinoma cells or control cells. (C) Left panel, schematic representation of T cells migrated to the lower chamber. Right panel, the absolute number and relative number of T cells in the lower chamber.

T cells were a major component of infiltrating cells in cancer stroma, which served as an inhibitor for cancer progression. To investigate the role of PHF6 in T cells activation and infiltration in endometrial carcinoma, the T cells were isolated from the healthy donors, then expanded and cocultured with PHF6 KD endometrial carcinoma cells or control cells in vitro (Figure 3A, left panel). We found that the proliferation of T cells cocultured with endometrial carcinoma cells was significantly increased when compared with T cells alone, while the growth of T cells was not changed in cocultured with PHF6 KD endometrial carcinoma cells group and cocultured with control endometrial carcinoma cells group (Figure 3A, right panel). We further detected the proliferating cell nuclear antigen (PCNA) in T cells when cocultured with HEC‐1‐A cells, KLE cells or T cells alone by flow. We found that endometrial carcinoma cells could increase the PCNA expression in T cells (Figure S3A). The IFN‐γ^+^ and TNF‐α^+^ T cells are active T cells in peripheral blood and bone marrow. To investigate whether PHF6 KD endometrial carcinoma cells could influence the activity of T cells, we analysed the IFN‐γ and TNF‐α expression in T cells when coculture with PHF6 KD or control endometrial carcinoma cells by flow cytometry. We found that the percentage of IFN‐γ^+^ and TNF‐α^+^ cells was increased in T cells when cocultured with PHF6 KD or control endometrial carcinoma cells, compared with T cells alone. However, the IFN‐γ and TNF‐α expression was similar in T cells cocultured with PHF6 KD and T cells cocultured with control endometrial carcinoma cells. It suggested that PHF6 did not influence the activation of T cells. To investigate the effect of PHF6 on T cells infiltration into cancer stroma, T cells and endometrial carcinoma cells (HEC‐1‐A and KLE cells) were cocultured using the double‐chamber. We observed the increased migration of T cells when cocultured with endometrial carcinoma cells, as compared to T cells alone in the lower chambers (Figure S3B). We observed the increased migration of T cells when cocultured with PHF6 KD endometrial carcinoma cells compared with control cells in the lower chambers (Figure 3C and Figure S3C). These results suggested that endometrial carcinoma cells could promote the proliferation and activation of T cells. Knockdown of PHF6 in endometrial carcinoma cells promoted the migration of T cells in UCECs.

### 3.4 PHF6 inhibits the infiltration of T cells by decreasing the IL32 expression in endometrial carcinoma cells

**FIGURE 4:**
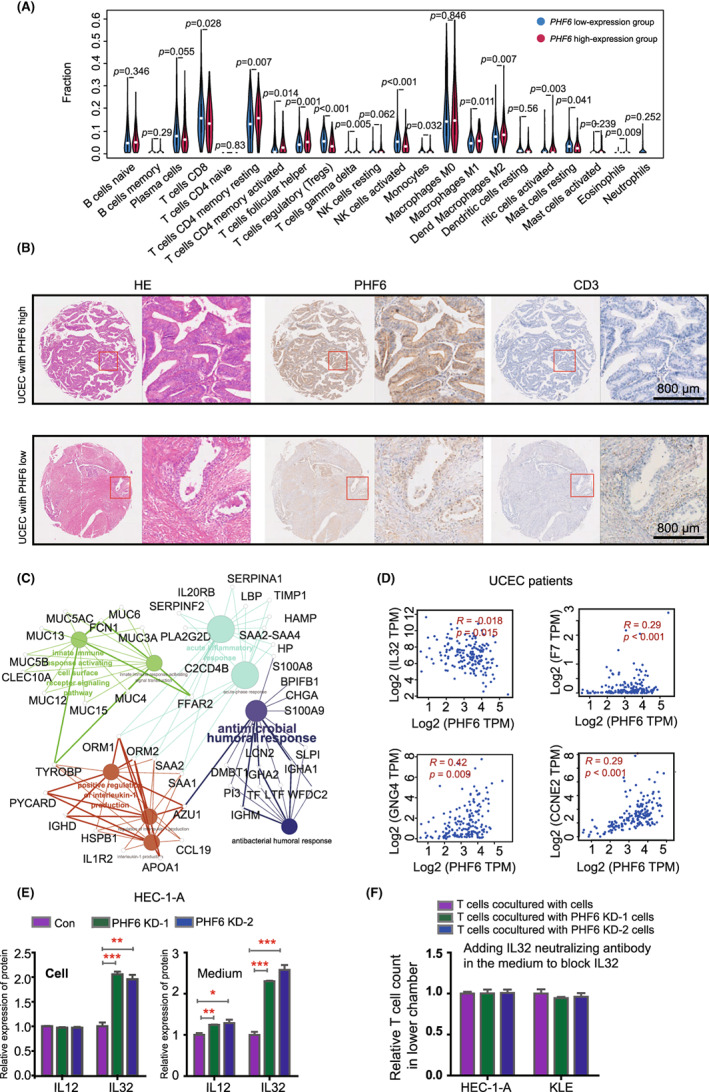
PHF6 inhibited T‐cell infiltration by decreasing the expression of IL32 in UCEC patients. (A) Wilcoxon rank‐sum test revealed the difference in the infiltration levels of 22 immune cells in the low‐PHF6 and the high‐PHF6 UCEC patients. (B) Immunohistochemistry (IHC) verified the correlation between the expression of PHF6 and the infiltration of CD3^+^ T cells in UCEC tissues. (C) The network of differentially expressed genes between the low‐PHF6 and the high‐PHF6 UCEC patients. (D) The relationship between the expression of PHF6 and immune‐related factors, such as IL32, F7, GNG4 and CCNE4. (E) Left panel, the protein level of IL12 and IL32 in PHF6 KD HEC‐1‐A and control cells by ELISA assay. Right panel, the protein level of IL12 and IL32 in the medium of PHF6 KD HEC‐1‐A and control cells by ELISA assay. (F) IL‐32 neutralizing antibody was added in the medium of PHF6 KD HEC‐1‐A cells, PHF6 KD KLE cells and control cells. The absolute number of T cells in the lower chamber was counted. The relative number of T cells in the lower chamber was evaluated.

To further investigate whether PHF6 participates in immune infiltration in UCEC patients, we compared the profiles of infiltrated immune cells between 269 high‐PHF6 and 269 low‐PHF6 UCEC patients from TCGA. We found that the infiltration levels of immunocytes that inhibited tumour progression, such as CD8^+^ T cells, CD4^+^ T cells, activated NK cells and M1 macrophages were much lower in the high‐PHF6 patients than in the low‐PHF6 patients (Figure 4A). The infiltration level of M2 macrophages, which promoted tumour progression, was higher in UCEC patients with high PHF6 expression than in UCEC patients with low PHF6 expression (Figure 4A). These results were validated in a TMA cohort by Immunohistochemistry (IHC) assay. We analysed the correlation between the protein level of PHF6 and infiltration of T cells, B cells and macrophages in UCEC patients. We found UCEC patients with high PHF6 expression had lower infiltration of CD3^+^ /CD4^+^ /CD8^+^ T cells than UCEC patients with low PHF6 expression (Figure 4B, Figure S4A, and Table S2). However, the IHC assay showed that few CD19^+^ B cells and CD33^+^ macrophages/monocytes infiltrated in UCEC tissues (Figure S4B,C), indicating that CD19^+^ B cells and CD33^+^ macrophages/monocytes were not the major components of infiltrating immunocytes in UCECs. Furthermore, we detected the CDK4 expression in UCEC patients from TMA cohort. We found that the correlation between the protein level of PHF6 and CDK4 was not significant in UCEC patients from TMA cohort (Figure S4D and Table S3). This was probably in the limited number of patients in TMA (*n* = 34).

To investigate the underlying molecular mechanism by which PHF6 regulated the migration of T cells in UCEC patients, we analysed the transcriptional profiles of PHF6‐high patients and PHF6‐low patients. A total of 679 DEGs were acquired by setting the threshold values to |log2‐fold‐change (FC)| > 1.0 and adjusted *p*‐value <0.05, which contain 170 upregulated genes and 509 downregulated genes (Figure S4E). Gene Ontology analysis revealed that the DEGs shared multiple overlapping enriched immune‐associated pathways, such as acute inflammatory response (*p* = 1.0E−04), innate immune response activating cell surface receptor signalling pathway (*p* = 3.4E−02), positive regulation of interleukin‐1 production (*p* = 1.4E−02) and antimicrobial humoral response (*p* = 1.8E−02) (Figure 4C and Figure S4F). In addition, we found that the expression of PHF6 was correlated with the expression of immune‐related and cell cycle‐related factors, such as IL32, F7, CD4 and CDK4 in UCEC patients (Figure 4D and Figure S4G,H). It has been reported that IL2, IL12, IL15, IL21 and IL32 played important roles in activation and infiltration of T cells. We further analysed the mRNA expression of IL2, IL12, IL15, IL21 and IL32 in PHF6 KD HEC‐1‐A cells and control cells. We found that PHF6 KD significantly increased the mRNA expression of IL12 and IL32 in endometrial carcinoma cells (Figure S4I). The protein level of IL32 was much higher in the medium of PHF6 KD HEC‐1‐A cells and PHF6 KD KLE cells than that of control cells, while the protein expression of IL12 was slightly increased in the medium of PHF6 KD HEC‐1‐A cells as compared with that of control cells (Figure 4E and Figure S4J,K), suggesting IL32 might be a candidate target factor of PHF6 in UCEC cells. To further evaluate the role of IL32 in T‐cell infiltration (Figure 3C), we added the IL‐32 neutralizing antibody in the medium of PHF6 KD HEC‐1‐A cells, PHF6 KD KLE cells or control cells to block IL‐32 signalling pathway (Figure S4L). Then, we detected the migration of T cells to cancer cells. We found that the PHF6 KD‐induced increased T‐cell infiltration was rescued by IL32 neutralizing antibody (Figure 4F and Figure S4L), suggesting that PHF6 might regulate T‐cell infiltration through IL32 signalling pathway in UCECs.

## 4 DISCUSSION

Cancer is caused by a progressive series of genetic and epigenetic alterations that transform normal cells into malignant cells.^2^, ^9^, ^10^ These alterations might be used as hallmarks for identifying tumour cells in clinical samples, further increasing the possibility of detecting malignant tumours at their early stages, assessing the disease extent and predicting clinical outcomes.^11^ It has been reported that alterations in PHF6, a highly conserved epigenetic transcriptional regulator, are key epigenetic mechanisms associated with leukaemia initiation and progression.^12^, ^13^, ^14^ The status of PHF6 in solid tumours remains elusive. In this study, we described the distinct expression patterns of PHF6 in tumours and normal tissues by using comprehensive pan‐tissue and pan‐cancer analysis of RNA‐Seq data from TCGA and investigated the association between the expression of PHF6 and immunocyte infiltration in UCEC patients. We identified that PHF6 expression was associated with the clinical outcomes of UCEC patients and was related to immunocyte infiltration in UCEC, which suggested that PHF6 might be a promising prognostic biomarker and participate in immune regulation.

PHF6 is involved in chemical chromatin modifications and plays a critical role in the tight regulation of gene expression during tissue homeostasis and development. Therefore, its alterations result in various diseases, such as tumours.^15^, ^16^ Somatic PHF6 mutations were first described in T‐ALL patients.^13^, ^17^ Loss of PHF6 promoted T‐ALL progression.^18^, ^19^ In contrast with PHF6's role as a tumour suppressor in T‐ALL, decreased PHF6 expression prolonged the overall survival of AML patients.^20^ In B‐cell acute lymphoblastic leukaemia (B‐ALL) models, knockdown of PHF6 significantly reduced B‐ALL cell proliferation.^18^, ^19^ In HeLa cells, knockdown of PHF6 inhibited cancer cell growth and delayed cell cycle.^21^ Consistent with the role of PHF6 in HeLa cells, we found that knockdown of PHF6 significantly reduced the growth of endometrial carcinoma cells by blocking cell cycle, while PHF6 KD did not influence the invasion ability of endometrial carcinoma cells (data not shown). Our study and previous studies suggest that PHF6, a double‐edged sword in tumours, can promote tumour progression or act oppositely to prevent tumour occurrence. PHF6 likely participates in complicated signalling networks that are tissue‐specific.

PHF6 is a highly conserved epigenetic transcriptional regulator that is important for embryonic development. It contains two imperfect PHD‐like zinc finger domains, two nuclear localization signals as well as a nucleolar localization sequence. PHF6 functions in chromatin‐mediated regulation of the gene expression. It directly binds with double‐stranded DNA via its atypical PHD2 domain.^22^ Co‐immunoprecipitation experiments revealed that PHF6 interacts with constituents of the NuRD complex, including CHD4, HDAC1 and Rbb4,^23^, ^24^ which suggested that it might implicate in nucleosome positioning and activating/repressing the target genes. In current study, we found that PHF6 affected the CDK4 and IL32 expression through transcriptional regulation in endometrial carcinoma cells. However, we did not see the changes which were related to epigenetic regulation in PHF6 KD cells. The underlying mechanism needs to be further investigated in the future study. High expression of CDK4 could promote the proliferation of endometrial carcinoma cells in vivo.^25^, ^26^ Abemaciclib, a selective CDK4 inhibitor, significantly inhibited endometrial carcinoma cell growth in the nude mice model.^25^, ^26^ Consistent with previous studies, we found that depletion of PHF6 significantly decreased CDK4 expression and inhibited endometrial carcinoma cell proliferation in vitro and in vivo. Additionally, CDK4 OE could promote the growth of PHF6 KD HEC‐1‐A cells (Figure 2I,J), suggesting that PHF6 might regulate the tumour growth through CDK4 signalling pathway. In addition, the Boyden chamber transwell assay showed that PHF6 KD did not influence the invasion ability of HEC‐1‐A cells (data not shown), suggesting that PHF6‐CDK4 axis might not involve in the tumour cell migration in UCECs.

Here, we displayed the expression landscape of PHF6 in tumour samples and normal samples of 33 cancers. We found that the expression of PHF6 was significantly higher in tumour samples than in normal samples in 15 cancers. A high expression level of PHF6 was associated with an unfavourable prognosis in UCEC, indicating that PHF6 might promote the initiation and progression of UCEC. Furthermore, UCEC patients with *PHF6* mutations had favourable survival compared with UCEC patients with WT *PHF6*, indicating that targeting PHF6 might be a potential therapeutic strategy for UCEC patients. The role of PHF6 in UCEC was validated by molecular and cell biology experiments. Depletion of PHF6 effectively inhibited the proliferation of endometrial carcinoma cells, which suggested that PHF6 might be a candidate therapeutic target for UCEC patients.

During the development of cancer, the interaction between cancer cells and the immune microenvironment is dynamic, and this process is regulated by a complicated signalling network.^27^ We further studied the role of PHF6 in shaping the tumour immune microenvironment in UCEC because (i) high expression of PHF6 occurred in UCEC tissues (Figure 1); UCEC patients with PHF6 mutations or UCEC patients with lower expression of PHF6 had a favourable prognosis (Figure 1); and (ii) the expression level of PHF6 may reflect the status of T cells infiltration in UCEC (Figures 3 and 4). In the T‐cell migration assay, we found that the PHF6 KD endometrial carcinoma cells could promote the infiltration of T cells, which further indicated that PHF6 played an essential role in tumour immune microenvironment in UCEC patients. In Bunpei Nabekis' and Li Hans' studies, they reported that IL32 promoted development and infiltration of Tregs in ESCC microenvironment.^28^, ^29^ In Alessandra Vultaggios' study, they reported that IL32 receptors expressed on CD8^+^ T cells, and IL32 involved in the differentiation and infiltration of CD8^+^ T cells.^30^, ^31^, ^32^ We proposed that the lower infiltration of both CD4^+^/CD8^+^ T cells and Tregs in PHF6‐high UCECs probably was due to the lower level of IL32 in PHF6‐high UCEC patients (Figure 4D).

Cancer immunotherapy involves artificial increasing the cytotoxic capacity of immune cells and stimulation of the immune cells to treat tumour cells.^33^, ^34^ In recent years, immunotherapy has demonstrated significant efficacy across various tumours, becoming a new therapeutic strategy in cancers such as UCEC.^35^ However, only a small proportion of patients benefit from this treatment strategy, and models or biomarkers to predict immunotherapy response are insufficient. More efficient biomarkers and novel approaches are urgent to discover for cancer patients. In UCEC, high expression of PHF6 decreased the infiltration of CD8^+^ T cells, CD4^+^ T cells, activated NK cells and M1 macrophages, which prevented tumour formation via immune surveillance, indicating that PHF6 might be a valuable marker for estimating the abundance of immune cells in UCEC. The percentage of CD8^+^ T cells in tumour tissues has been reported as an independent predictor of increased OS and disease‐free survival (DFS) in uterine corpus endometrial carcinoma patients.^36^ Hence, the enrichment of effective immunocytes in endometrial cancers may lead to more favourable treatment outcomes for UCEC patients.^37^, ^38^ Consistently, our study found that high expression of PHF6 contributed to lower effective immunocyte infiltration and was associated with a worse prognosis of UCEC patients. These data also supported the conclusion that a higher infiltration of immunocytes is necessary for the immune system to target tumours efficiently.^38^ It is tempting to speculate that blocking PHF6 may enhance the immunotherapy response in endometrial cancers.

Investigation of the biological mechanisms and functions of oncogenes and tumour suppressors involved in tumorigenesis is an effective approach to achieve personal and precise treatment for tumour patients. Our work provides new insights into the underlying molecular mechanisms and functions of the epigenetic gene PHF6 in tumorigenesis. PHF6 might be a candidate prediction biomarker that may serve as an additional indication for immunotherapies and other anticancer strategies in UCEC. Prospective studies are needed to investigate the correlation between PHF6 expression and immunotherapy response. It is essential to better understand the underlying mechanisms of PHF6 in the tumour immune microenvironment in UCEC.

## AUTHOR CONTRIBUTIONS

**Xiaomin Wang:** Conceptualization (lead); data curation (lead); formal analysis (lead); funding acquisition (equal); investigation (lead); writing – original draft (lead). **Aizhong Fang:** Data curation (equal); formal analysis (equal); methodology (equal); software (equal). **Yichen Peng:** Data curation (equal); validation (equal); writing – original draft (equal). **Jianyu Yu:** Methodology (equal); validation (equal); visualization (equal). **Chunna Yu:** Data curation (equal); methodology (equal). **Jinxuan Xie:** Data curation (equal); formal analysis (equal); methodology (equal); software (equal). **Yi Zheng:** Data curation (equal); resources (equal). **Lairong Song:** Project administration (equal); supervision (equal); visualization (equal). **Parker Li:** Writing – original draft (equal); writing – review and editing (equal). **Jia Li:** Methodology (equal); project administration (equal); supervision (equal); validation (equal); visualization (equal). **Xun Kang:** Methodology (equal); resources (equal); visualization (equal). **Yi Lin:** Project administration (lead); supervision (equal); visualization (equal); writing – review and editing (equal). **Wenbin Li:** Funding acquisition (equal); supervision (lead); writing – review and editing (equal).

## FUNDING INFORMATION

This work was supported by funds from Beijing Postdoctoral Science Foundation (2021‐ZZ‐019 to X. Wang); the National Natural Science Foundation of China (82070169 to X. Wang & 81972338 to W. Li).

## CONFLICT OF INTEREST

The authors declare no conflict of interest.

## Supporting information


Appendix S1

Appendix S2

Appendix S3

Appendix S4

Appendix S5

Appendix S6

## Data Availability

All data and materials in the current study are available.
