# Supplementary material for: PHF6 promotes the progression of endometrial carcinoma by increasing cancer cells growth and decreasing T‐cell infiltration
Source: J Cell Mol Med. 2023 Feb 8;27(5):609–21. doi: 10.1111/jcmm.17638 (PMC9983320; doi:10.1111/jcmm.17638)
Supplement: Supplementary file 1 — Appendix S1 [file JCMM-27-609-s002.docx]

**Supplementary materials: four figures and three tables.**

**Supplementary figure 1.** **Low expression of PHF6 or *PHF6* mutation predicted the favorable clinical outcomes of UCEC patients.** (A) PHF6 mRNA expression in different cancers and their paired normal tissues from the TCGA and GTEx database. (B) The frequency of *PHF6* mutations in UCEC patients from the TCGA database.


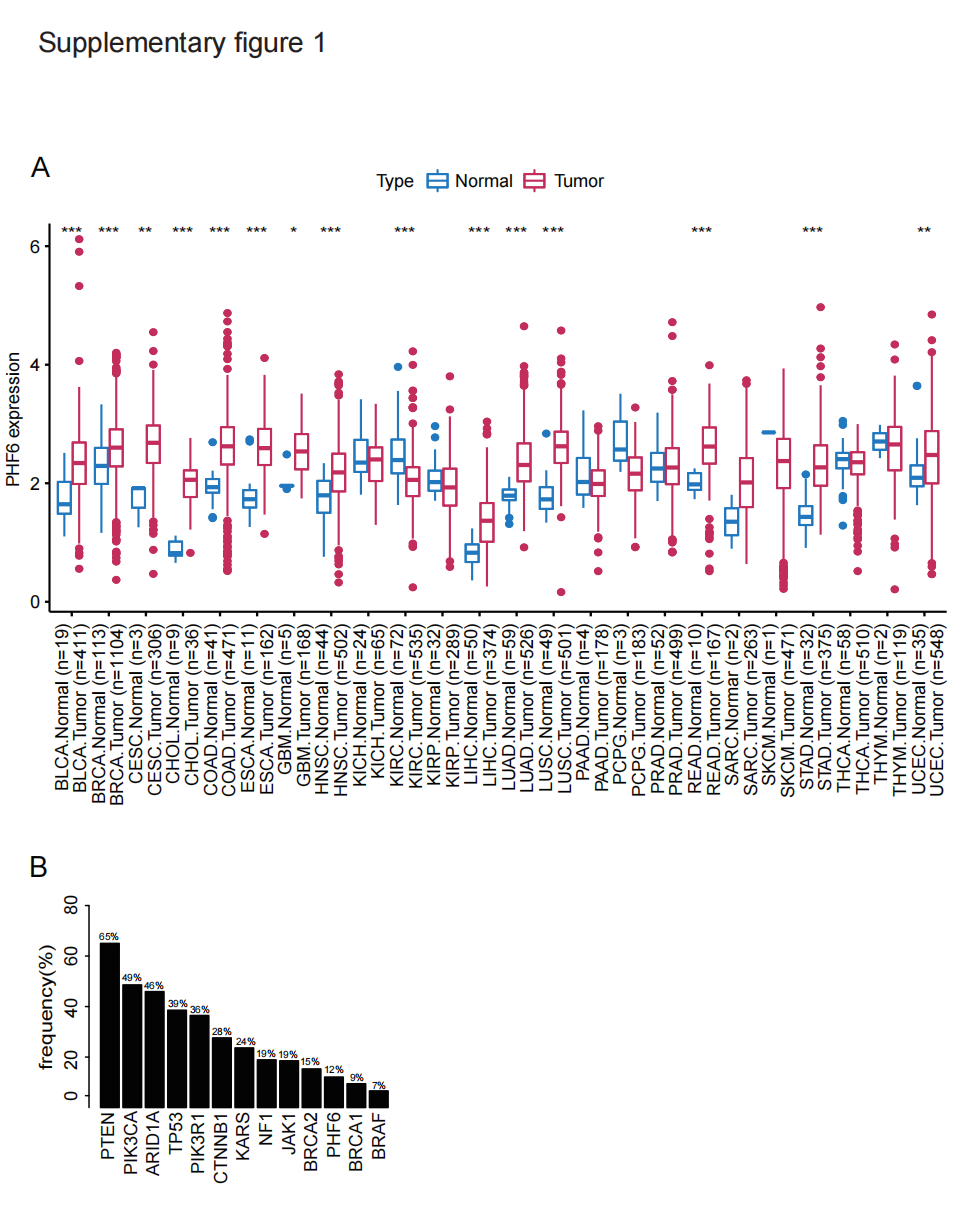


**Supplementary figure 2.** **Knock down of PHF6 inhibited the growth of endometrial carcinoma cells through blocking cell cycle in *vitro* and in *vivo*.** (A) The mRNA expression of PHF6 in PHF6 KD endometrial carcinoma cells and control cells. (B) The cell cycle stage of PHF6 KD HEC-1-A cells, PHF6 KD KLE cells, and control cells by Ki67 and Hochest 33342 staining. (C-D) The cell cycle stage of PHF6 KD HEC-1-A cells and control cells by PI staining. (E) The apoptosis level of PHF6 KD endometrial carcinoma cells and control cells. (F) The mRNA expression of CDK1-4 and CDK6-7 in HEC-1-A cells. (F) The mRNA expression of PHF6, CDK4, and CDK7 in PHF6 KD endometrial carcinoma cells and control cells. (G) The protein expression of PHF6 and CDK4 in tumors from mice implanted with PHF6 KD HEC-1-A cells or control cells.


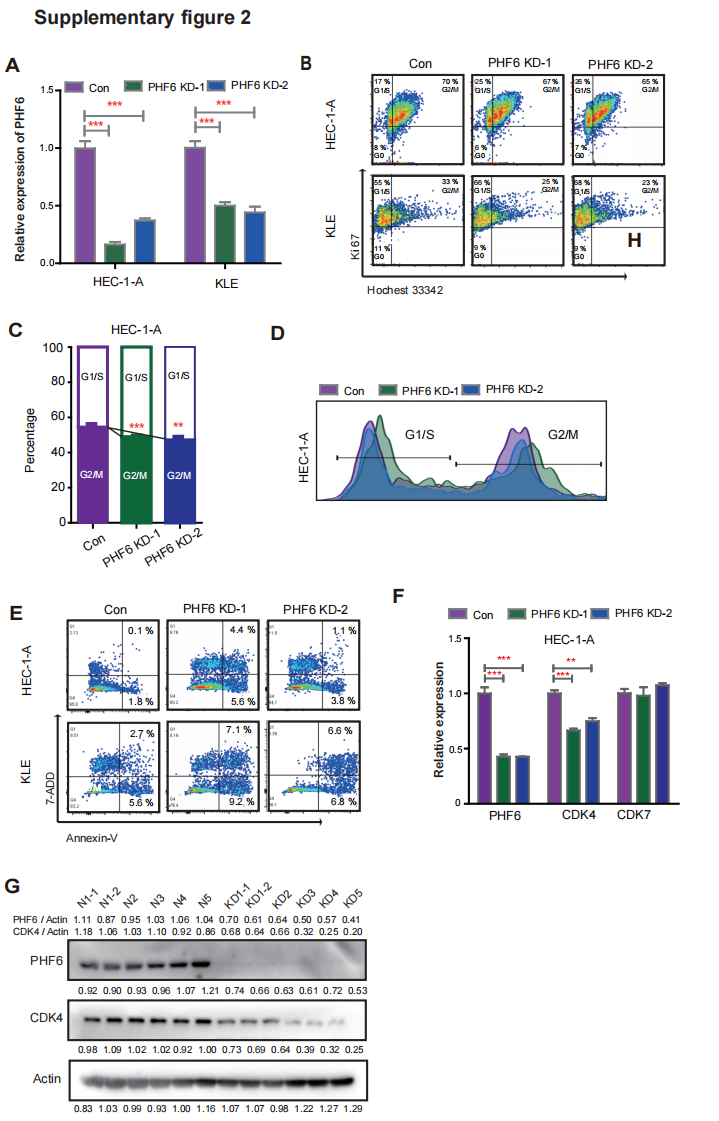


**Supplementary figure 3.** **Knock down of PHF6 in endometrial carcinoma cells promoted T cells migration.** (A) The PCNA expression in T cells when co-cultured with KLE cells, HEC-1-A cells or T cells alone. (B) T cells were co-cultured with KLE cells, HEC-1-A cells or T cells alone using the double-chamber. The absolute number and relative number of T cells in the lower chamber. (C) T cells were co-cultured with PHF6 KD KLE cells, PHF6 KD HEC-1-A cells or control cells using the double-chamber. The pictures showed the T cells on the lower membrane surface.


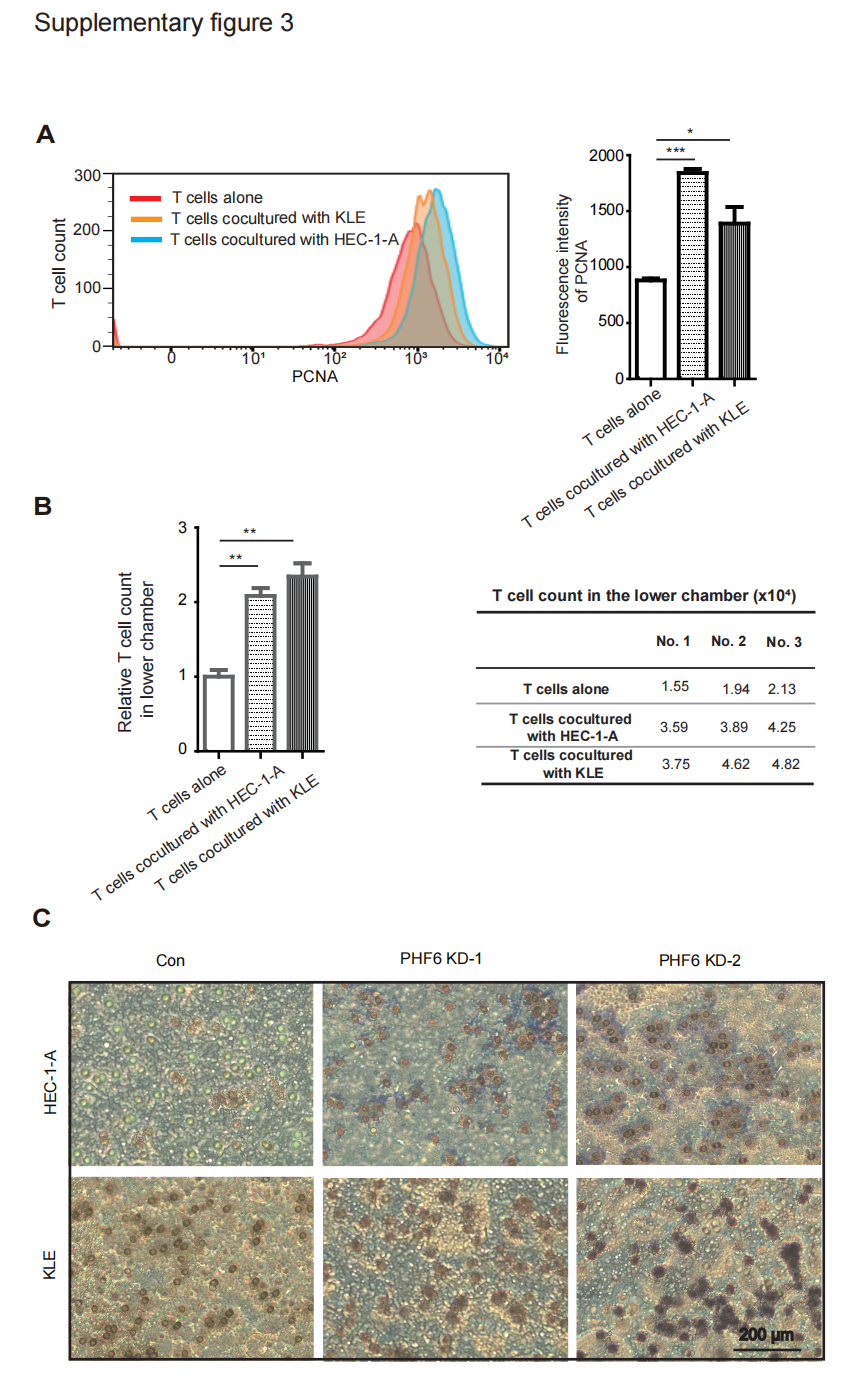


**Supplementary figure 4. PHF6 inhibited T cells infiltration through decreasing the expression of IL32 in UCEC cells.** (A) Immunohistochemistry (IHC) showed the infiltration of CD4^+^ T cells and CD8^+^ T cells in UCEC tissues. (B-C) IHC showed the infiltration of CD19^+^ B cells and CD33^+^ macrophages/monocytes in UCEC tissues. (D) The protein expression of CDK4 in UCEC patients with PHF6 high expression and UCEC patients with PHF6 low expression from TMA cohort by IHC assay. (E) Volcano plot: differentially expressed genes between UCEC patients with high PHF6 and UCEC patients with low PHF6. (F) The dot plot for the cellular component, molecular function, and biological process data in GO analysis in UCECs. (G) The relationship between the expression of PHF6 and CD4. (H) The relationship between the expression of PHF6 and CDK4. (I) The mRNA expression of IL2, IL12, IL15, IL21 and IL32 in PHF6 KD HEC-1-A or control cells. (J) The protein level of IL12 and IL32 in PHF6 KD KLE and control cells by ELISA assay. (K) The protein level of IL12 and IL32 in the medium of PHF6 KD KLE and control cells by ELISA assay. (L) IL-32 neutralizing antibody was added in the medium of PHF6 KD HEC-1-A cells, PHF6 KD KLE cells, and control cells. The absolute number of T cells in the lower chamber was counted. The relative number of T cells in the lower chamber was evaluated.


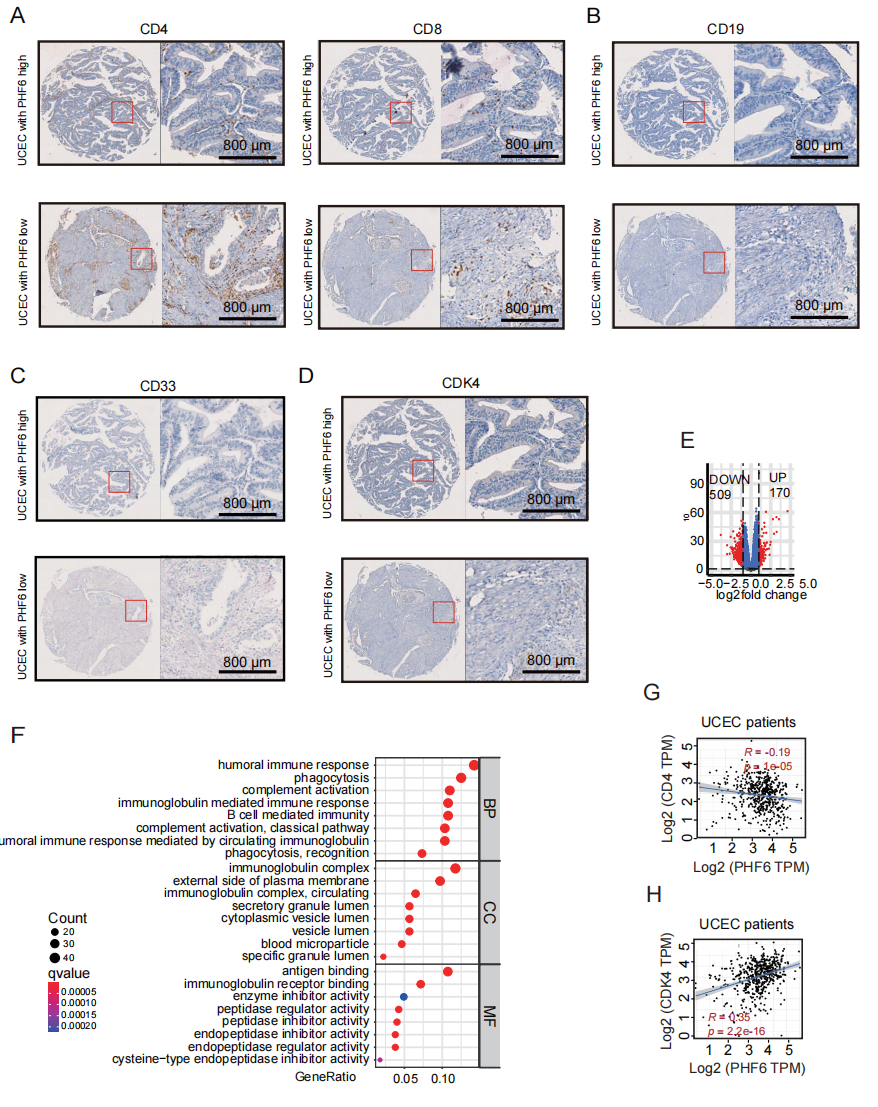


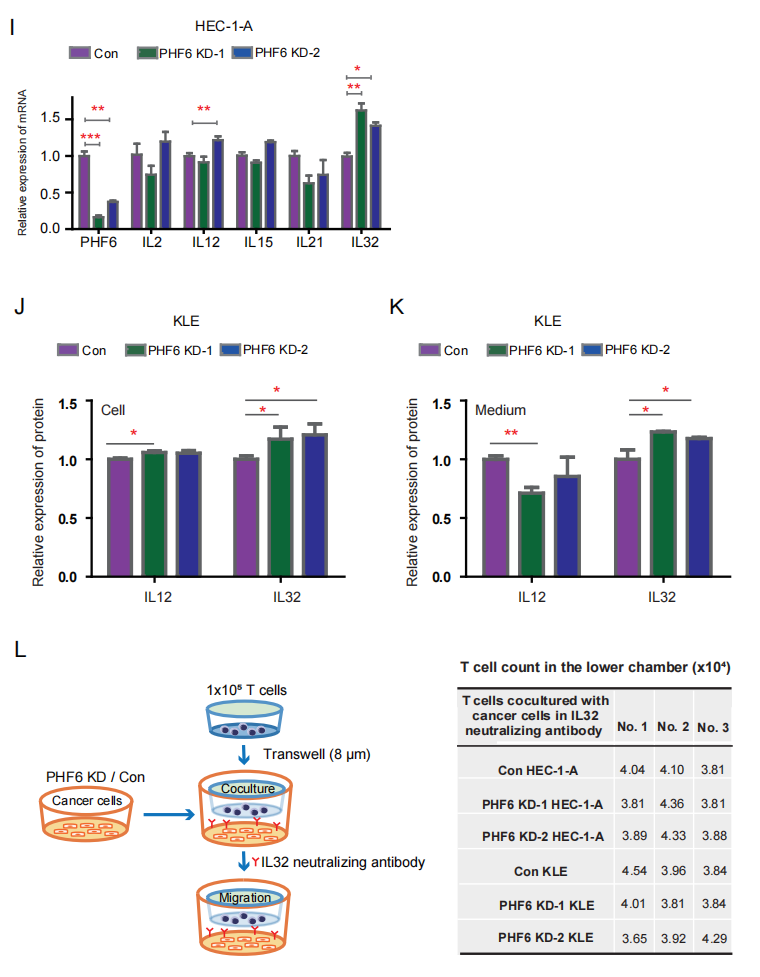


| **Supplementary table 1.** Correlation of PHF6 Expression and Clinicopathological Features in UCECs from TCGA database | | | |
| --- | --- | --- | --- |
|  | UCEC | | *p* value |
| PHF6 | high | low |  |
| **Age(years),n(%)** | 541 | |  |
| <65 | 147 | 141 | 0.525 |
| ≥65 | 137 | 116 |  |
| **Gender,n(%)** | 544 | |  |
| Male |  |  |  |
| Female | 285 | 259 |  |
| **clinical_stage,n(%)** | 544 | |  |
| Stage I | 172 | 168 | 0.706 |
| Stage II | 28 | 23 |  |
| Stage III | 70 | 54 |  |
| Stage IV | 15 | 14 |  |
| **histological_grade,n(%)** | 544 | |  |
| G1 | 37 | 61 | 0.0004（***） |
| G2 | 56 | 64 |  |
| G3 | 188 | 127 |  |
| G4 | 4 | 7 |  |
| **tumor_status,n(%)** | 523 | |  |
| TUMOR FREE | 216 | 205 | 0.369 |
| WITH TUMOR | 58 | 44 |  |

**Supplementary table 2.** The relationship between PHF6 expression and CD3^+^ T cell infiltration.

|  |  | PHF6 high (45) | PHF6 low (21) |
| --- | --- | --- | --- |
| Percentage of CD3^+^ cell | ≦40% | 29 (64%) | 12 (57%) |
|  | >40% | 16 (36%) | 9 (43%) |

|  |  | PHF6 high (45) | PHF6 low (21) |
| --- | --- | --- | --- |
| Percentage of CD3^+^ cell | ≦40% | 26 (58%) | 12 (57%) |
|  | >40% | 19 (42%) | 9 (43%) |

**Supplementary table 3.** The relationship between PHF6 expression and CDK4 expression in UCEC patients in TMA cohort.

|  | CDK4 | | *p* value |
| --- | --- | --- | --- |
| PHF6 | high | low | 0.7425 |
| high | 11 | 20 |  |
| low | 4 | 10 |  |

|  | CDK4 | | *p* value |
| --- | --- | --- | --- |
| PHF6 | high | low | 0.08715 |
| high | 19 | 12 |  |
| low | 4 | 10 |  |
